# Analysing Sr isotopes in low‐Sr samples such as single insects with inductively coupled plasma tandem mass spectrometry using N_2_O as a reaction gas for in‐line Rb separation

**DOI:** 10.1002/rcm.8604

**Published:** 2020-02-05

**Authors:** David Thomas Murphy, Charlotte M. Allen, Osama Ghidan, Andrew Dickson, Wan‐Ping Hu, Ethan Briggs, Peter W. Holder, Karen F. Armstrong

**Affiliations:** ^1^ School of Earth, Environmental and Biological Sciences Queensland University of Technology Brisbane Queensland Australia; ^2^ Institute for Future Environments Queensland University of Technology Brisbane Queensland Australia; ^3^ School of Biological Sciences University of Queensland Brisbane Queensland Australia; ^4^ Bio‐Protection Research Centre Lincoln University Lincoln New Zealand

## Abstract

**Rationale:**

Strontium isotopes are valuable markers of provenance in a range of disciplines. Limited amounts of Sr in low‐mass samples such as insects mean that conventional Sr isotope analysis precludes their use for geographic origins in many ecological studies or in applications such as biosecurity. Here we test the viability of using inductively coupled plasma tandem mass spectrometry (ICP‐MS/MS) with N_2_O as a reaction gas for accurately determining Sr isotopes in insects with Sr < 100 ng.

**Methods:**

Strontium isotopes were determined in solution mode using ICP‐MS/MS with 0.14 L/min N_2_O as a reaction gas to convert Sr^+^ into SrO^+^ for in‐line separation of ^87^Sr from ^87^Rb. The Sr isotope reference standards NIST SRM 987, NIST SRM 1570a and NIST SRM 1547 were used to assess accuracy and reproducibility. Ten insect species collected from the wild as a proof‐of‐principle application were analysed for Sr concentration and Sr isotopes.

**Results:**

Using ICP‐MS/MS we show for the first time that internal mass bias correction of ^87^Sr^16^O/^86^Sr^16^O based on ^88^Sr^16^O/^86^Sr^16^O works to give for NIST SRM 987 a ^87^Sr/^86^Sr ratio of 0.7101 ± 0.012 (RSD = 0.17%) and for NIST SRM 1570a a ^87^Sr/^86^Sr ratio of 0.7100 ± 0.009 (RSD = 0.12%), which are within error of the accepted values. The first ^87^Sr/^86^Sr ratio of NIST SRM 1547 is 0.7596 ± 0.0014. Strontium analyses were run on 0.8 mL of 0.25–0.5 ppb Sr, which equates to 2–4 ng of Sr. Strontium isotope analysis with a precision of >99.8% can be achieved with in‐line separation of ^87^Sr from ^87^Rb at least up to solutions with 25 ppb Rb.

**Conclusions:**

A minimum of 5 mg of insect tissue is required for Sr isotope analysis. This new ICP‐MS/MS method enables Sr isotope analysis in single insects, allowing population‐scale studies to be feasible and making possible applications with time‐critical uses such as biosecurity.

## 1 INTRODUCTION

Here we test a newly developed solution method for strontium (Sr) isotope analysis using inductively coupled plasma tandem mass spectrometry (ICP‐MS/MS), after the work of Bolea‐Fernandez et al^1^ for its suitability in assessing the provenance of very small quantities of biological material of biosecurity relevance. Based on the high spatial variation of ^87^Sr/^86^Sr in the geosphere and the fact that Sr is expected to be present in relatively high concentration in many biological tissues,^2^, ^3^ Sr isotopes are recognised as powerful tracers of provenance.^4^, ^5^, ^6^, ^7^ The geographic variability of bioavailable Sr isotope ratios is related to a combination of their inherent variation in different bedrock geologies and the Sr being from a variety of environmental and anthropogenic sources.^8^, ^9^, ^10^, ^11^ Importantly, there is no evidence for fractionation of Sr isotopes during biological processes.^12^, ^13^

The appreciably high quantities of Sr in many biological tissues allows for straightforward Sr isotope analysis using conventional methods such as thermal ionisation mass spectrometry (TIMS) and multi‐collector ICP‐MS. However, prior to this current study, the use of ^87^Sr/^86^Sr as a provenancing tool had not been possible where only a very low amount of sample was available. Consequently, there has been only rare consideration of Sr isotopes for insect provenancing,^14^ and that case was driven by the urgency to know the geographic origins of high‐risk plant pests where there was typically only one or very few specimens. The small mass of such species/samples means that the quantity of Sr available for isotope analysis is commonly suboptimal (<100 ng of Sr) for conventional methods. This requires pooling of multiple specimens, a demand that would make interpretation of provenance challenging. For biosecurity samples this is further compounded by the need to subsample individual specimens for other techniques, such as DNA species identification. In contrast, the ICP‐MS/MS method^1^ requires significantly lower quantities of Sr (2–4 ng) than conventional methods, allowing individual specimens to be analysed. The compromise is lower precision, *ca* 0.2% relative standard deviation (RSD),^1^ but relative to the degree of variation involved with geographic provenancing this is not as crucial a factor as for many geological applications. The method also has a much faster turnaround time in the order of days rather than weeks, because the isobaric interference between ^87^Rb and ^87^Sr can be removed directly during analysis. This is achieved using the reaction gas N_2_O, where Sr^+^ reacts with N_2_O and is converted to SrO^+^, while Rb^+^, which is in a noble gas electron configuration, does not react. This chemical behaviour allows the Sr isotope ratios to be calculated from the Sr^16^O^+^ ions and the additional laborious chemical separation step required for conventional Sr isotope analysis is eliminated.

Here, for the first time, the stability and accuracy of ^87^Sr/^86^Sr isotope analyses using the ICP‐MS/MS method with N_2_O as a reaction gas are investigated. In doing so we also assess the quantity of Sr required for analysis with the ICP‐MS/MS method. Secondly, we investigate issues related to Sr isotope detection limits, specifically the Sr concentration and the quantity of Sr present in wild‐caught specimens of different insect species, to determine the minimum mass of insect tissue required. Finally, we trial the application of this method for determining the ^87^Sr/^86^Sr isotope composition of insects from the metropolitan Brisbane area (Queensland, Australia), to illustrate its use for gathering baseline reference data of bioavailable Sr isotopes for future insect provenance studies.

## 2 EXPERIMENTAL

The experimental strategy is to use a tandem mass spectrometer with a flow of reactive N_2_O gas into the reaction cell, in order to mass‐shift ^87^Sr away from the isobarically interfering ^87^Rb. One quadrupole allows a selected mass into the reaction cell that may or may not react with N_2_O, and a second quadrupole chooses what mass to allow access to the detector. In this way the host of possible interferences on the Rb–Sr isotope system, including the isobaric interference of Rb and Sr at mass 87, is minimised. Complications can include interference of Kr from the carrier gas, K, Ca and Ti dimers, and doubly charged heavy rare earth elements on Sr. Whereas these molecules and more highly charged species can interfere on single‐quadrupole instruments, they cannot get through the double mass filtering provided by the tandem quadrupole (MS/MS) set‐up unless they undergo an analogous reaction to Sr.

The tandem ICP mass spectrometer used in this study was a Thermo iCap TQ ICP‐MS instrument (Thermo Fisher Scientific, Bremen, Germany). N_2_O was preferred as a reaction gas over O_2_, SF_6_ or CH_3_F because Hogmalm et al^15^ observed that N_2_O provided the highest yield of SrO^+^. Consequently, high‐purity N_2_O (ULSI 5N; Matheson Tri‐Gas Inc., Basking Ridge, NJ, USA) was plumbed into the O_2_ gas line for the purpose of this experiment. The sample introduction system was an MVX‐7100 Microlitre Workstation autosampler from Teledyne Cetac Technologies (Omaha, NE, USA). This autosampler uses one syringe to pump the sample solution up into a user‐defined (in this case 0.8‐mL) loop and a second syringe to push the sample solution into the nebuliser, in this case at a rate of 100 μL/min.

The general operating conditions of the ICP‐MS instrument, the isotopes analysed and the acquisition parameters are listed in Table 1. Daily tuning of the instrument was first performed in single‐quadrupole (SQ) mode using the interface tune option in the Qtegra software (Thermo Fisher Scientific) and then in triple‐quadrupole (QqQ) mode using the software advanced tune option in MS/MS mode to optimise the Bi counts. There were nine analytical sessions. Sensitivity values for ^85^Rb and ^88^Sr in SQ mode and ^85^Rb and ^88^Sr^16^O in MS/MS mode are presented in Table 1.

**Table 1:** ICP‐MS instrument settings and sensitivities in pulse counting for SQ (MS) mode and QqQ (MS/MS) mode

Parameter	Value
**Instrument settings**	
RF power	1550 W
Ar nebuliser flow rate	1.13 L/min
Ar auxiliary gas flow rate	0.8 L/min
Ar cooling gas flow rate	14 L/min
Sampling depth	5.70 mm
	
**Sensitivities**	
SQ mode CeO^+^/Ce^+^	1.8%
SQ mode Ce^2+^/Ce^+^	4.7%
QqQ mode Ce^+^/SQ mode Ce^+^	2.6%
	
SQ mode ^88^Sr cps/0.1 ppb	520 000
QqQ mode ^88^Sr cps/0.1 ppb	260 000
SQ mode ^85^Rb cps/0.1 ppb	130 000
QqQ mode ^85^Rb cps/0.1 ppb	58 000

Pulse/analogue counter crossover *ca* 1.5 million counts, detector cross‐calibrated daily.

In view of the potentially complex sample matrix (i.e. digested whole insects), we opted for a thorough approach to analyte selection. The best‐possible isotope ratio precision would be achieved by spending all the dwell time on the only three relevant masses ^86,87,88^Sr^16^O. However, this would leave the analysis vulnerable to potential artefacts arising from interferences. To safeguard against this issue, we measured the ^84,86,87,88^Sr masses, the ^84,86,87,88^Sr^16^O masses and the ^85,87^Rb masses, as well as ^83^Kr^16^O, to monitor the most likely interferences on masses 102, 103 and 104. The isotopes of Ru and Pd (with masses of 102 and 104) were not monitored as these masses would not get through the first quadrupole which was allowed to range between masses 84 and 88. The dwell time for each mass analysed was 0.025 s, except for ^83^Kr^16^O that had a dwell time of 0.01 s. Ten replicates each consisting of 170 sweeps yielded a total analysis time of 6:40 minutes. The analysis time was set to fit within the 8 minutes of sample introduction into the ICP mass spectrometer, defined by the 0.8‐mL loop of the MVX‐7100 Microlitre Workstation and the rate of sample introduction of 100 μL/min. In terms of the three masses (^86,87,88^Sr^16^O masses) on which the counting statistics for the final mass bias‐corrected ^87^Sr/^86^Sr depend, each replicate was analysed for 12.75 s, i.e. 127.5 s for an individual sample.

Three standard reference materials were analysed: NIST SRM 987, NIST SRM 1547 and NIST SRM 1570a (National Institute of Standards and Technology, Gaithersburg, MD, USA). NIST SRM 987 is a strontium carbonate and Sr isotope standard (^87^Sr/^86^Sr 0.71034 ± 0.0003) in wide use. It was diluted from a stock solution in double‐distilled 2% HNO_3_ for analysis. The NIST standards SRM 1570a ‘spinach leaves’ (^87^Sr/^86^Sr 0.70905 ± 0.00002)^16^ and SRM 1547 ‘peach leaves’ were dissolved in concentrated double‐distilled HNO_3_, dried down and the residue dissolved in 2% HNO_3_ to make stock solutions that could be diluted for analysis. Building on the work of Bolea‐Fernandez et al^1^ who used CH_3_F as a reaction gas and who observed that Sr isotope measurements with an Agilent (Santa Clara, CA, USA) 8800 ICP‐MS/MS instrument required external mass bias correction using sample standard bracketing with NIST SRM 987, we systematically analysed interleaved NIST SRM 987 and 1570a throughout all the analytical sessions.

Individual insect specimens were dried overnight at 40°C. Any surface contaminants were then blown off with a gentle flow of Ar gas^17^ prior to the specimens being accurately weighed using a four‐decimal‐place balance. The insects were placed in either quartz or glass 15‐mL digestion vessels and 1 mL of concentrated double‐distilled HNO_3_ was added to each digestion vessel, as it also was to the procedural blanks. The samples and blanks were then placed in a Milestone bench‐top Ultra‐wave single reaction chamber microwave digestion system (Milestone Srl, Sorisole, Italy), with an external chamber microwave‐absorbing base solution of 25°C and 40 Bar. The digestions were performed at a microwave power of 1500 W with temperature increase up to 220°C at 100 Bar in 15 minutes. The temperature and pressure were kept constant at 220°C and 100 Bar, respectively, for another 10 minutes before the samples were cooled down. After cooling and decompression, the samples were transferred to pre‐cleaned and pre‐weighed 10‐mL polypropylene centrifuge tubes with sufficient Milli‐Q water (Millipore, Burlington, MA, USA) added to generate minimum 10‐mL stock solutions. These tubes were then weighed again to determine the concentration.

For trace element concentration determination, including Sr, aliquots of each sample were added to pre‐cleaned 1.8‐mL polypropylene vials, diluted 10 times using 2% HNO_3_ with a 0.9 ppb indium spike added as an internal standard. The solutions were run in the iCap TQ ICP mass spectrometer in single‐quadrupole mode following a standard multi‐element analysis procedure. The USGS rock standard W2 (US Geological Survey, Reston, VA, USA) was repeat analysed as a calibration standard based on the procedures of Eggins et al.^18^

## 3 SR ISOTOPE ANALYSIS USING ICP‐MS/MS

### 3.1 Optimising N_2_O

**Figure 1:**
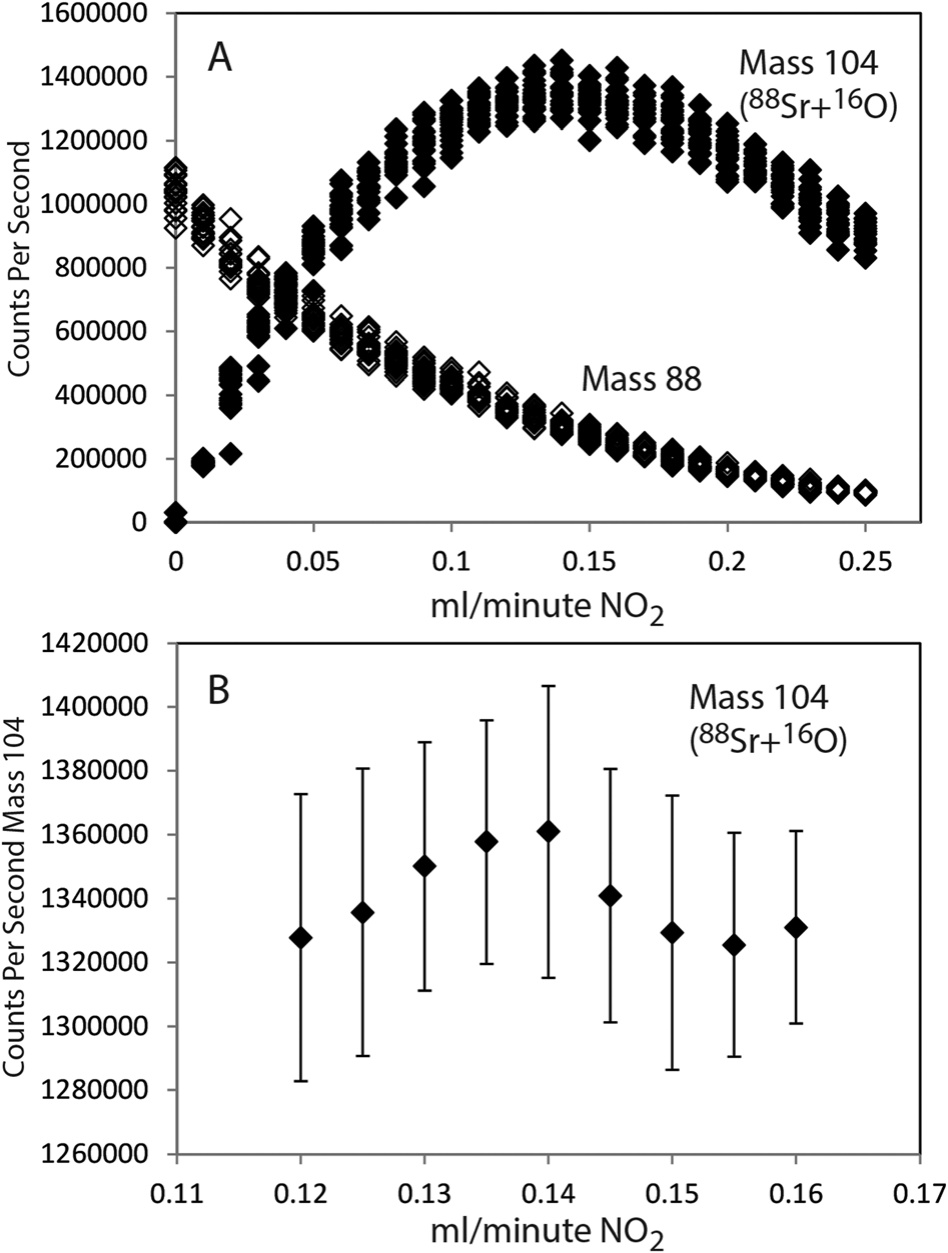
Optimising SrO^+^ yield by monitoring cps of both ^88^Sr and ^88^Sr^16^O. A, Relative change in SrO^+^ yield by monitoring cps of both ^88^Sr and ^88^Sr^16^O as the flow rate of N_2_O was varied from 0 to 2.5 mL/min. B, Yield of ^88^Sr^16^O plateaus between 0.12 and 0.16. Note that at the maximum yield of ^88^Sr^16^O there is still considerable ^88^Sr

To optimise the production of Sr oxide in the reaction chamber, the flow rate of N_2_O was adjusted and the effect on a solution of NIST SRM 987 was examined. The dilution of the reference material was introduced into the ICP mass spectrometer to give 1 million counts per second (cps) on mass 88, ^88^Sr, in MS/MS mode at 0 mL/min N_2_O. Analysing in time‐resolved mode, the flow rate of N_2_O was steadily increased from 0 to 0.25 mL/min in intervals of 0.01 mL/min. Figure 1 shows that ^88^Sr gradually decreases throughout the experiment. By contrast, ^88^Sr^16^O increases rapidly initially then reaches a plateau between 0.12 and 0.18 mL/min N_2_O and finally starts to decrease at higher flow rates. A further fine‐scale experiment between 0.12 and 0.18 mL/min N_2_O indicated that the ideal flow rate was 0.14 mL/min N_2_O (Figure 1). For all other experiments, the dilution level targets were between 0.5 and 1 million cps on ^88^Sr^16^O, which equates to 0.25 to 0.5 ppb Sr in the solutions.

The higher yield of ^88^Sr^16^O than of ^88^Sr at 0 mL/min N_2_O reflects the effect of tuning in MS/MS mode, which optimises the sensitivity of Bi with N_2_O in the collision cell. A noteworthy observation is that even at the highest N_2_O flow rate there remained unreacted ^88^Sr, with the optimised N_2_O flow rate leaving about 30% of the Sr as unreacted. The analytical strategy is to maximise SrO counts, not to achieve the highest reaction rate (SrO/Sr).

### 3.2 Assessing the influence of Rb on Sr and RbO on SrO

**Figure 2:**
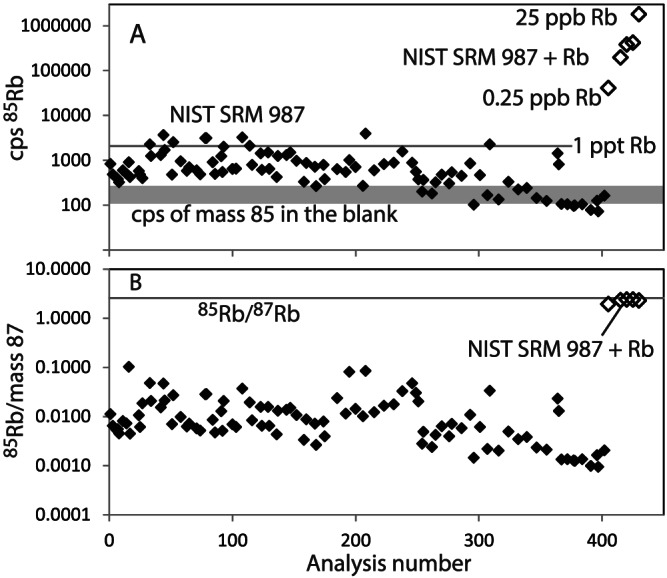
Measurements of Rb and Sr for NIST SRM 987(*n* = 96) and NIST SRM 987 doped with 0.25 to 25 ppb Rb (*n* = 5) over a MS/MS analytical session for unreacted isotopes. A, cps of ^85^Rb that were used to represent the Rb signal as there are no significant isotopic interferences with ^85^Rb. The trend shows a reduction in the Rb blank in near‐new plastic wear during progressive analysis. B, ^85^Rb/mass 87 where mass 87 represents a mixture of ^87^Rb and unreacted ^87^Sr and varies depending on the Rb/Sr ratio of the sample. The horizontal line represents the natural ^85^Rb/^87^Rb of 2.593. The NIST SRM 987 ^85^Rb/mass 87 is much lower than the natural ^85^Rb/^87^Rb because the measurement is dominated by unreacted ^87^Sr. The Rb‐doped NIST SRM 987 ^85^Rb/mass 87 approaches that of the natural ^85^Rb/^87^Rb (1.9–2.4)

For viable Sr isotope analysis with on‐line separation of Rb from Sr, instead of the historical method of chemically separating them prior to analysis, one must demonstrate that Sr oxide production is efficient and that Rb oxide production is negligible. The reaction cell method is based on measuring the ratios of the Sr oxide species with the assumption that RbO^+^ is not an interference at mass 87 + 16 = 103. Analysis of the highly pure Sr carbonate, NIST SRM 987, diluted to 0.2 ppb Sr and doped with Rb (0.025 to 25 ppb Rb; Table 2) is used to highlight the effect of the isobaric interference of Rb on Sr and RbO on SrO by comparison with undoped NIST SRM 987 (Figure 2).

**Table 2:** Raw Sr isotope ratios and mass bias‐corrected Sr isotope ratios using different mass bias laws for NIST SRM 987, with one standard deviation errors (1 s) and relative standard deviation (RSD)

Raw data	Ratio	1 s	RSD (%)
Raw ^87^Sr/^86^Sr	0.7432	0.12	15.79
Raw ^87^Sr/^86^Sr plus 0.25 ppb Rb	3.4839		
Raw ^87^Sr/^86^Sr plus 2.5 ppb Rb	14.2442		
Raw ^87^Sr/^86^Sr plus 25 ppb Rb	139.1110		
Raw ^87^Sr^16^O/^86^Sr^16^O	0.7275	0.0022	0.31
**Mass bias correction**	^**87**^ **Sr** ^**16**^ **O/** ^**86**^ **Sr** ^**16**^ **O**	**1 s**	**RSD (%)**
Linear correction	0.7101	0.0012	0.17
Exponential correction	0.7093	0.0011	0.16
Power‐law correction	0.7095	0.0011	0.16
Sample standard bracketing	0.7101	0.0018	0.25
	^**87**^ **Sr/** ^**86**^ **Sr**	**1 s**	**RSD (%)**
Reference value	0.7103	0.0003	0.04

Mass 85, ^85^Rb, was monitored because this Rb mass is free of the Sr interference and can be used to predict the number of counts produced by ^87^Rb because of the known natural relative abundances of ^85^Rb/^87^Rb. Ideally, these Rb ions enter the collision cell and transition through without undergoing reaction, to be detected at mass 85 (and mass 87). The undoped NIST SRM 987 analyses yielded between 70 and 3000 cps ^85^Rb on nine analytical days and, based on the doping experiments, this represents concentrations of <2 ppt Rb (Figure 2A). For the Rb‐doped NIST SRM 987, the solution with 0.025 ppb Rb yielded 41 000 cps ^85^Rb and the aliquot with 25 ppb Rb produced 1 801 000 cps ^85^Rb.

The ^85^Rb/mass 87 ratio provides an indication of the effect of the isotopic overlap between ^87^Rb and ^87^Sr, albeit from the Rb viewpoint, and why separation of the two elements is mandatory for accurate ^87^Sr/^86^Sr measurement. For the NIST SRM 987 analyses, this ratio is consistently below 0.1 as the ratio is dominated by unreacted ^87^Sr because of the low Rb concentration in this Sr reference material (Figure 2B). In contrast, the ^85^Rb/mass 87 ratios for the Rb‐doped NIST SRM 987 (1.9–2.4) are much higher and dominated by Rb, with slightly lower ratios than the natural unfractionated ^85^Rb/^87^Rb isotope ratio of 2.593 because of unreacted ^87^Sr.

**Figure 3:**
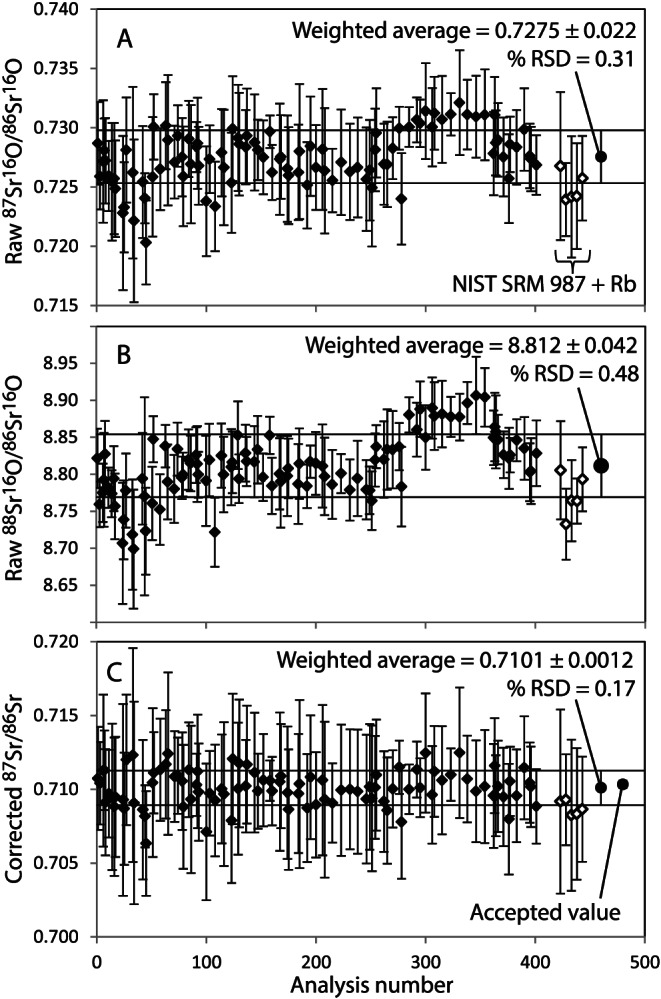
Raw count ratios of A, ^87^Sr^16^O/^86^Sr^16^O and B, ^88^Sr^16^O/^86^Sr^16^O of measured NIST SRM 987. C, Results of ^88^Sr^16^O/^86^Sr^16^O mass bias‐corrected ^87^Sr^16^O/^86^Sr^16^O using a linear law. The average ^87^Sr/^86^Sr ratios for NIST SRM 987 (*n* = 96) and NIST SRM 987 doped with 0.025–25 ppb Rb are within accepted values. The horizontal lines represent the one standard deviation on NIST SRM 987 calculated from all data

For the undoped NIST SRM 987, the raw ^87^Sr/^86^Sr ratio is 4.6% higher than the reference value (Table 1), with a 16% RSD reflecting the low but variable Rb present in the solutions evident from the ^85^Rb (Figure 2A). The raw ^87^Sr/^86^Sr ratios of the Rb‐doped NIST SRM 987 are markedly higher (Table 2). Despite the high Rb concentrations for the five Rb‐doped NIST SRM 987 measurements, the raw ^87^Sr^16^O/^86^Sr^16^O is indistinguishable from the weighted average of 96 repeat analyses of NIST SRM 987 (Figure 3A). While this does not conclusively demonstrate the absence of RbO^+^ formed by reaction of Rb^+^ and N_2_O in the collision cell, it does indicate that there is no resolvable effect on ^87^Sr^16^O in mixed solutions containing 0.2 ppb Sr and up to 25 ppb Rb.

### 3.3 ^87^Sr/^86^Sr stability and accuracy determined from ^86,87,88^Sr^16^O

The results for NIST SRM 987 are presented in Figures 3A and 3B, where we show 96 repeat determinations over the nine analytical sessions. These are of the raw counts of ^87^Sr^16^O/^86^Sr^16^O and ^88^Sr^16^O/^86^S^16^O. The results for the five Rb‐doped NIST SRM 987 analyses are also presented. Each datum is shown with its one standard deviation repeatability error. Also included are the weighted average and the standard deviation of the weighted average. The five Rb‐doped NIST SRM 987 measurements are all within error of the weighted average for both raw ^87^Sr^16^O/^86^Sr^16^O and ^88^Sr^16^O/^86^Sr^16^O. Similar to the observations of Bolea‐Fernandez et al^1^ the raw ratios are biased high: 2.42% higher for our ^87^Sr^16^O/^86^Sr^16^O compared with the 2.53% positive bias for the ^87^SrF/^86^SrF of Bolea‐Fernandez et al^1^ who used CH_3_F as a reaction gas.

**Figure 4:**
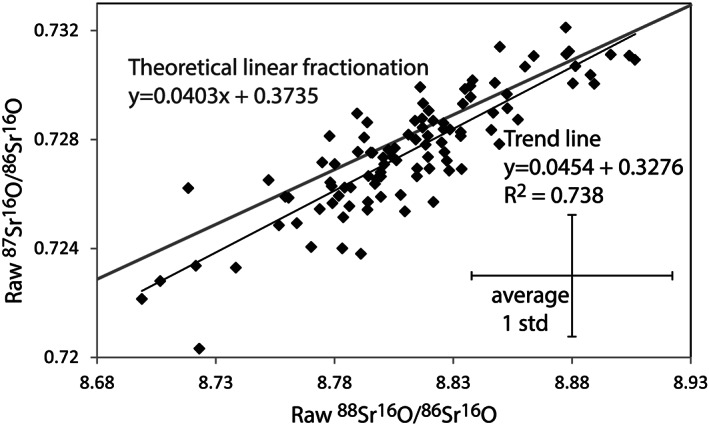
The linear correlation of raw counts of ^88^Sr^16^O/^86^Sr^16^O and ^87^Sr^16^O/^86^Sr^16^O for NIST SRM 987 (*n* = 96). The average one standard deviation repeatability error is also shown for the dataset. The grey line represents the theoretical linear fractionation between masses ^86^Sr^16^O, ^87^Sr^16^O and ^86^Sr^16^O

Our data show very strong correlation between the raw ^87^Sr^16^O/^86^Sr^16^O and ^88^Sr^16^O/^86^Sr^16^O ratios and all measurements are within error of theoretical linear fractionation trend (Figure 4A). Both the exponential and the power‐law fractionation trends plot at slightly higher ^87^Sr^16^O/^86^Sr^16^O and ^88^Sr^16^O/^86^Sr^16^O (not shown) and are not within error of all measurements. Therefore, the linear fractionation law is preferred for mass bias correction.

Figure 3C shows a plot of the results of using ^88^Sr^16^O/^86^Sr^16^O and a linear fraction to correct for mass bias of the ^87^Sr^16^O/^86^Sr^16^O to give the corrected ^87^Sr/^86^Sr and its weighted average, standard deviation of the weighted average and the accepted value for the reference material. The average value is indistinguishable from the certified value. All measurements, including those of the five Rb‐doped NIST SRM 987 samples, are within error of the weighted average. Corrections with the exponential or power laws overcorrect the ^87^Sr/^86^Sr (Table 2), albeit still within error of the reference value. If sample standard bracketing is used for mass bias correction with the adjacent ^87^Sr^16^O/^86^Sr^16^O NIST SRM 987 measurements, an identical corrected ^87^Sr/^86^Sr is obtained but with a higher error.

Note that no change in the linear fractionation factor for mass bias correction was observed over the range of N_2_O flow rates used for the SrO^+^ yield optimisation experiment.

The finding that internal mass bias correction using the sample's natural ^88^Sr^16^O/^86^Sr^16^O ratio yields fully corrected and accurate ^87^Sr/^86^Sr ratios contrasts with the observation of Bolea‐Fernandez et al^1^ who found that the ^88^SrF/^86^SrF measurements using an Agilent 8800 ICP‐MS/MS instrument only partially corrected the ^87^SrF/^86^SrF (0.62%) and an additional external mass bias correction using NIST SRM 987 for sample standard bracketing was required. No such external mass bias correction was required for our data. The second difference concerns precision. Bolea‐Fernandez et al^1^ achieved an RSD of 0.04%, compared with the 0.17% of the present study. This reflects counting statistics as that study^1^ used a longer duty cycle per replicate for the ^86,87,88^SrF and did not analyse any other masses. At a similar duty cycle per replicate, the precision is similar to that of Figure 2 of Bolea‐Fernandez et al.^1^

The results for NIST SRM 1570a spinach leaves (Rb/Sr by weight = 0.23, raw ^87^Sr/^86^Sr = 5.7) and NIST SRM 1547 peach leaves (Rb/Sr = 0.37, raw ^87^Sr/^86^Sr = 8.0) are presented in Table 3. The corrected ^87^Sr/^86^Sr weighted average for NIST SRM 1570a is just outside the standard deviation of the three TIMS measurements reported by DeBord et al.^16^ As the long‐term external errors of ^87^Sr/^86^Sr TIMS measurements are higher than the internal error of a single study, we are confident that our weighted average for NIST SRM 1570a will be within error of composites of high‐precision analyses. That the mass bias‐corrected ^87^Sr/^86^Sr measurements for the Rb‐bearing NIST SRM 1570a are all within error of the accepted values indicates that RbO^+^ had no measurable effect.

**Table 3:** Sr isotope ratio results for plant standards

Mass bias correction	^87^Sr^16^O/^86^Sr^16^O	1 s	RSD (%)
NIST RM 1570a spinach leaves (*n* = 52)			
Raw data	0.7282	0.0019	0.26
Linear correction	0.7100	0.0009	0.12
Sample standard bracketing	0.7105	0.0017	0.23
	^87^Sr/^86^Sr	1 e	RSD (%)
Reference value *n* = 3^16^	0.70905	0.00002	0.003
			
**Mass bias correction**	^**87**^ **Sr** ^**16**^ **O/** ^**86**^ **Sr** ^**16**^ **O**	**1** s	**RSD (%)**
NIST RM 1547 peach leaves (*n* = 36)			
Raw data	0.7784	0.0020	0.26
Linear correction	0.7596	0.0014	0.18
Sample standard bracketing	0.7587	0.0040	0.53

The corrected ^87^Sr/^86^Sr weighted average for NIST SRM 1547 is the first reported ^87^Sr/^86^Sr ratio for this reference standard. Also included in Table 2 is the sample standard bracketing correction for both plant standards using adjacent ^87^Sr^16^O/^86^Sr^16^O NIST SRM 987 measurements, which yields a higher error than internal linear correction.

Our results indicate that it is possible to achieve an accuracy of greater 99.8% on corrected ^87^Sr/^86^Sr in 0.8 mL of solution with Sr concentrations of between 0.25 and 0.5 ppb, which equates to 2–4 ng of Sr. This precision is not as high as can be achieved with multi‐collector ICP‐MS or TIMS. However, these methods require significantly more Sr and require prior separation of Rb from Sr. Multi‐collector ICP‐MS Sr isotope analysis requires 100–500 ppb Sr in 1.5 mL of solution (depending on the machine vintage), which equates to 150 to 500 ng of Sr, while TIMS Sr isotope analysis conventionally requires about 500 ng of Sr.

## 4 ASSESSING THE VIABILITY OF ANALYSING SR ISOTOPES ON SINGLE INSECTS

### 4.1 Sample collection

The insect samples listed in Table 4 were from known sites in the metropolitan Brisbane area (Appendix 1, supporting information). They were from dried pinned museum collections except for the sawflies and scale insects which were collected fresh. Only adults were used. This is the easiest life stage to identify to species, the dispersing life stage for which provenance is questioned for pest species, and in which Sr from the immature damaging (pest) life stage has accumulated from the diet at the place of origin. Factors that may impact on the levels of Sr available, and therefore on the ability to measure ^87^Sr/^86^Sr in individual insects, are the size of the insect and potentially different levels of Sr in different plant parts used for food. All species, except the Australian hornet (trophic level 3, mixed Sr sources in the diet), were plant feeders (trophic level 2, single plant Sr source) representing the different feeding guilds feeding on different plant parts. To consider the host plant as a potential source of Sr concentration variation is difficult as plant pests are typically polyphagous, with no way of knowing what the immature life stage of a caught adult would have fed upon. Not all examined species are considered pests. However, the shield bugs represent a feeding model for the brown marmorated stink bug, a high‐risk biosecurity species not yet established in Australia, and the leaf feeders also represent potential biosecurity risks in those insect orders.

**Table 4:** Insects used for ^87^Sr/^86^Sr analysis, with size and feeding habit considered as factors that might impact on Sr concentration in individual specimens

Order: Family	Species	Common name	Approx. Size (mm)	Primary feeding habit	Ref.
	(pest*)				
Coleoptera: Cerambycidae	Phoracantha semipunctata* (Fabricius 1775)	Eucalyptus long‐horned borer	14–30	Wood borer	Hanks et al^19^
Coleoptera: Scarabidae	Rhopaea magnicornis* (Blackburn 1888)	Brown cockchafer or rhopaea cane grub	21–30	Root feeder	Miller et al^20^
Hemiptera: Pentatomindae	*Theseus modestus* (Stal 1865)	Gum tree shield bug	14–16	Xylem feeders	Naumann^21^
	*Austromalaya reticulate* (Westwood 1837)	Brown long‐headed shield bug	13–18		
Hymenoptera: Apidae	*Amegilla cingulata* (Fabricius 1775)	Blue‐banded bee	14	Pollen, nectar	Leijs et al^22^
	*Apis mellifera* (Linnaeus 1758)	European honey bee	17		vanEngelsdorp et al^23^
Hymenoptera: Vespidae.	*Abispa ephippium* (Fabricius 1775)	Australian hornet	30	Carnivore	Matthews et al^24^
Lepidoptera: Nymphalidae	*Euploea core‐corinna* (Cramer 1780)	Common crow (butterfly)	95	Leaf feeder	Scheermeyer et al^25^
Hymenoptera: Pergidae	*Lophyrotoma* sp. (Ashmead 1998)	Sawflies	2.5–20	Leaf feeder	Burrows et al^26^
Hemiptera: Monophlebid	Icerya seychellarum* (Westwood 1855)	Seychelles scale insect	10	Phloem feeder	Unruh et al^27^

### 4.2 Sr concentration and quantity in different insects

**Figure 5:**
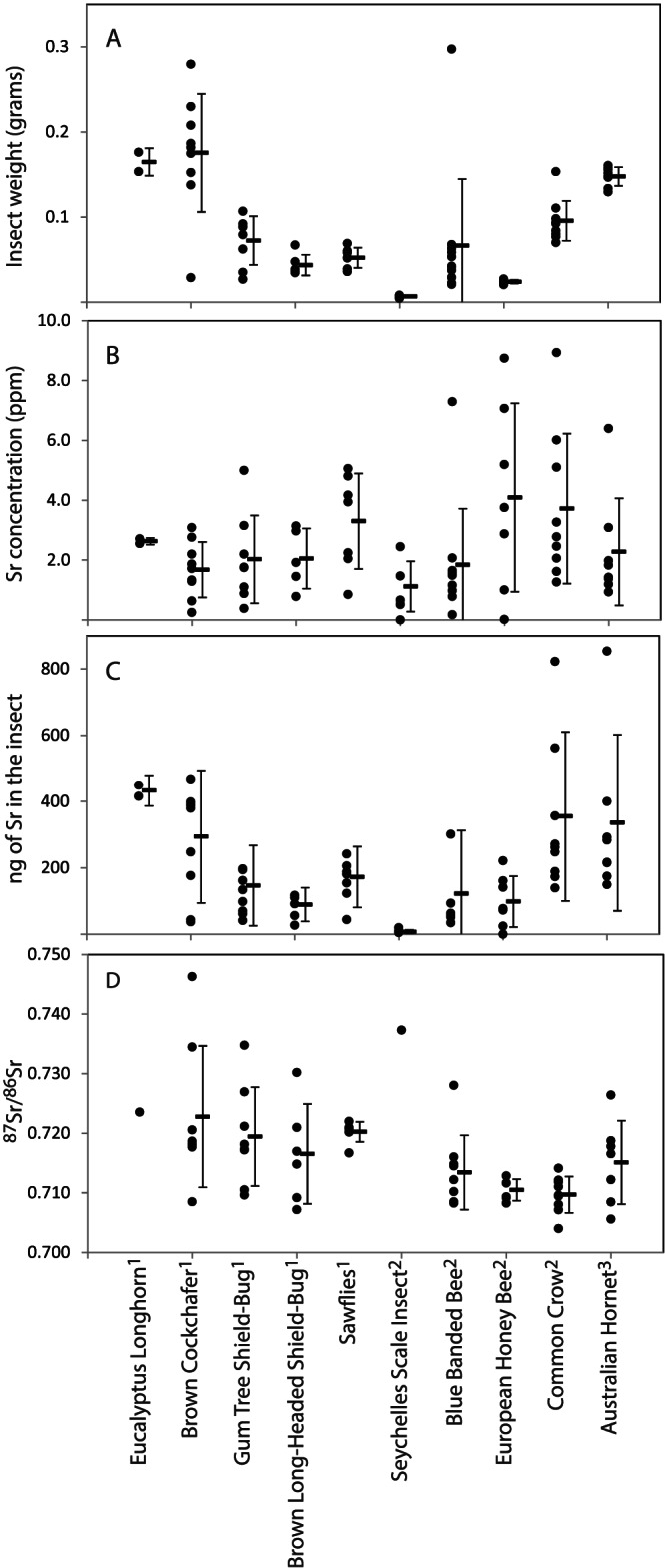
A, Plot of the variation in insect weight; B, Sr concentration; C,  quantity of Sr; and D, ^87^Sr/^86^Sr variation in each of the 10 different insect species analysed. Two samples with anomalously high Sr are not shown in (B) and (C), a brown long‐headed shield bug with 24 ppm Sr and a common crow butterfly with 22 ppm Sr. For each insect species the average and standard deviation are shown, again excluding the two anomalous samples in (B) and (C)

The insects vary in weight from the comparatively small Seychelles scale, with an average weight of 7 mg, to the more substantial brown cockchafer with an average weight of 175 mg (Table 5, Figure 5). The ranges of Sr concentrations for all insect species overlap and there is no obvious variation in Sr concentration among the different insect species. The principal control on the quantity of Sr available for isotope analysis is the mass of the insect (Figure 5). Most insects had greater than 20 ng of Sr (Table 5, Figure 5) with the exception of the Seychelles scale insects that had an average of 8 ng of Sr.

**Table 5:** Average weight, Sr concentration and quantity of Sr for each insect species

Common name	*n*	Mean Sr conc. (ppm)^a^	Standard deviation	Mean insect weight (mg)	Standard deviation	Sr (ng)	Error^b^
Eucalyptus long‐horned borer	2	2.63	0.11	165	16	433	46
Brown cockchafer	9	1.68	0.93	175	69	294	200
Gum tree shield bug	8	2.03	1.47	72	29	147	121
Brown long‐headed shield bug^c^	6	2.05	1.00	44	12	90	51
Blue‐banded bee	11	1.84	1.88	67	78	123	190
European honey bee	7	4.09	3.15	24	2	99	76
Australian hornet	8	2.27	1.79	148	11	336	266
Common crow^c^	10	3.72	2.50	96	23	356	255
Sawflies	7	3.30	1.59	52	12	172	92
Seychelles scale insect	7	1.12	0.84	6.8	1.5	8	6

a The Sr concentrations of all of the insects together with 42 additional elements are provided in Appendix 1 (supporting information).

b Propagated error from the standard deviations on the mean Sr concentration and insect weight.

c An insect with anomalously high Sr content has been excluded from calculating the average and standard deviation.

### 4.3 Measurement of Sr isotopes in insects

The ^88^Sr^16^O/^86^Sr^16^O, the mass‐shifted non‐radiogenic Sr isotopes pair, for most of the insect measurements was indistinguishable from the reference materials. However, a number of samples had anomalously high ^88^Sr^16^O/^86^Sr^16^O (>8.95, the greatest value measured for the reference materials) and thus were more than three standard deviations from the mean NIST SRM 987 measured value (Figure 3). A proportion of these insect samples were remeasured and the second measurement gave ^88^Sr^16^O/^86^Sr^16^O within three standard deviations of NIST SRM 987. For these remeasured samples, the first anomalously high ^88^Sr^16^O/^86^Sr^16^O measurement overcorrected the ^87^Sr^16^O/^86^Sr^16^O relative to that of the second measurement with typical ^88^Sr^16^O/^86^Sr^16^O. Using extraordinary isotope ratio as a reason to reject, seven samples that had ^88^Sr^16^O/^86^Sr^16^O > 8.95 and were unable to be remeasured have been excluded from the results. For the Seychelles scale insects with low Sr quantities, only one sample gave a reportable Sr isotope measurement.

### 4.4 Variation in ^87^Sr/^86^Sr in insects of different species and from different geographic sites

**Figure 6:**
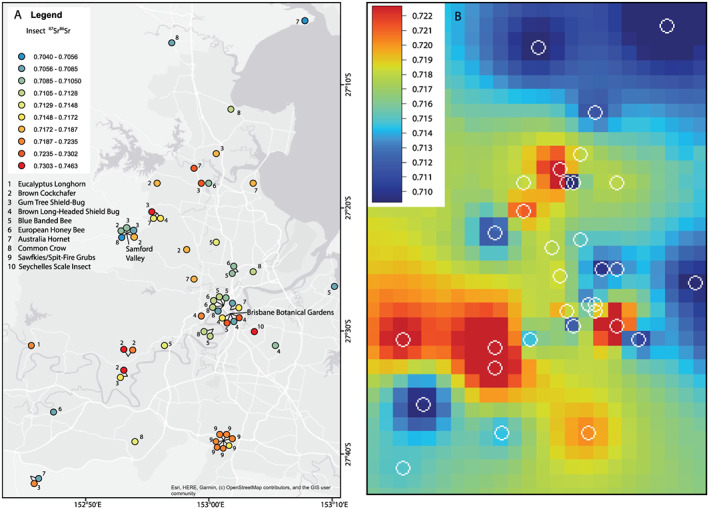
A, Geographical spread in the collected insects and their Sr isotope variation around metropolitan Brisbane. There are four samples from outside the map area. B, Interpolated Sr isotope variation in the shown map area using the insect Sr isotope data and an angular distance‐weighted (ADW) grid interpolation from irregular distributed points^28^ with a cell size of 0.02° resolution [Color figure can be viewed at wileyonlinelibrary.com]

The ^87^Sr/^86^Sr measured in the insects range from 0.7040 to 0.7462 (Figure 6). The insect Rb/Sr ranges from 0.11 to 31 with no significant correlation between ^87^Sr/^86^Sr and Rb/Sr. For nine duplicate measurements and three triplicate measurements, the measurements are within error and the standard deviations are lower than the internal precision of the measurements. Given this replicate population, the standard deviations are presented instead of the internal precision in Appendix 1 (supporting information).

The insect species that record the greatest variation in ^87^Sr/^86^Sr are those that have been collected over the widest geographic spread, such as the brown cockchafer (0.7228 ± 0.012) and the gum tree shield bug (0.7194 ± 0.008) (Figure 6A). Conversely, the insect species with the smallest variation in ^87^Sr/^86^Sr are the sawflies (0.7202 ± 0.002) which were all collected from the one sample site (Figure 6A). Interestingly the variation in ^87^Sr/^86^Sr in the European honey bee (0.7105 ± 0.002) and the common crow butterfly (0.7097 ± 0.003) is low considering the wide geographic spread of collection sites.

The relatively wide geographic range of sampling sites allows for an initial assessment of the geographic variability of Sr isotopes as recorded in the insects. Most localities from which two or more samples were acquired have variable Sr isotope ratios (Figure 6A). For example, insects collected from both the Brisbane Botanical Gardens and the Samford Valley record highly variable Sr isotopes (0.7072–0.7302 and 0.7040–0.7180, respectively). Despite this local heterogeneity, there are general spatial trends in the Sr isotope variation across metropolitan Brisbane that can be seen through data interpolation (Figure 6B) with regions of interpolated high ^87^Sr/^86^Sr commonly in close proximity to regions of interpolated low ^87^Sr/^86^Sr.

## 5 DISCUSSION

### 5.1 Mass bias correction and interferences

Our analysis of NIST SRM 987, Rb‐doped NIST SRM 987 and NIST SRM 1570a indicates that, as theory predicts, it is possible to internally correct for mass bias on ^87^Sr^16^O/^86^Sr^16^O with ^88^Sr^16^O/^86^Sr^16^O and that ^87^Rb^16^O has no effect on the ^87^Sr^16^O/^86^Sr^16^O using the Thermo iCap TQ ICP‐MS instrument with N_2_O as a reaction gas. It is unclear why successful internal mass bias correction using ^88^Sr^16^O/^86^Sr^16^O to correct for mass bias on ^87^Sr^16^O/^86^Sr^16^O works on the iCap TQ ICP‐MS instrument but is very consistently off by *ca* 2% on an Agilent 8800 ICP‐MS instrument.^1^ This probably relates to machine architecture differences between the two ICP instrument types.

However, beyond instrument difference, and focusing on the iCap TQ ICP‐MS instrument, a number of measurements of insects in solution had elevated ^88^Sr^16^O/^86^Sr^16^O (<8.95) relative to the standards. The most likely cause of the high^88^Sr^16^O/^86^Sr^16^O is an isobaric interference on mass 104. For such an interference to occur, an ion or charged molecule with a mass‐to‐charge ratio the same as that of ^88^Sr^+^ must be allowed through the first quadrupole; there it must react with ^16^O to give a mass‐to‐charge ratio the same as that of ^88^Sr^+16^O to get through the second quadruple to the detector. We are discussing specifically here the reason for elevated ^88^Sr^16^O/^86^Sr^16^O; however, the same logic applies to potential interferences on the other SrO^+^ species.

Doubly charged ^176^Yb, ^176^Lu and ^176^Hf will be allowed through the first quadrupole with ^88^Sr^+^. These doubly charged ions would then need to react with N_2_O to form doubly charged oxides to be allowed through the second quadruple to the detector. For the insects that had ^88^Sr^16^O/^86^Sr^16^O > 8.95, the concentrations of Yb, Lu and Hf (Appendix 1, supporting information), when above detection level, are one to three orders of magnitude lower than those of Sr. This requires the production yields of doubly charged oxides to be very high in order to affect mass 104, which is unlikely. This is because the concentrations of Yb, Lu and Hf for the insects with ^88^Sr^16^O/^86^Sr^16^O < 8.95 are indistinguishable from those for other insects analysed. Therefore, it is highly unlikely that doubly charged Yb, Lu and Hf oxides have a significant effect on ^88^Sr^16^O/^86^Sr^16^O.

Singly charged dimers ^*x*^M_1_^88−*x*^M_2_^+^ (e.g. ^44^Ca.^44^Ca^+^, ^40^Ca.^47^Ti^+^ and ^39^K.^48^Ti^+^) will be allowed through the first quadrupole and could react with N_2_O to form singly charged oxides (^*x*^M_1_^88−*x*^M_2_^16^O^+^) that will be allowed through the second quadrupole to the detector. The insects are expected to have high concentrations of biologically mediated Ca and K, while Ti ranges in concentration from 5 to 800 ppm (Appendix 1, supporting information). Therefore, even at low yields of ^*x*^M_1_^88−*x*^M_2_^16^O^+^, mass 104 could be affected. However, all insects are expected to have high Ca and K and the insects with ^88^Sr^16^O/^86^Sr^16^O > 8.95 have indistinguishable Ti concentration from those of other insects analysed.

The origin of the elevated ^88^Sr^16^O/^86^Sr^16^O remains unclear and further experimentation is required to assign the interferences.

### 5.2 Feasibility of measuring Sr isotopes in single insects

As a proof of principle, this new ICP‐MS/MS method was confirmed as making a valuable contribution to our ability to measure Sr isotopes in low‐mass biological specimens, specifically using a small test group of wild insects. The levels of Sr in individual specimens were, for the most part, useful for downstream isotope analysis although these insects covered a range of species sizes, habitats and diet sources. Each of those factors could have an influence on the levels of Sr in the individual specimens, but size appeared to be a likely limiting factor. Usefully, a simple correlation of insect versus Sr weights suggests that insects of less than 5 mg in weight are likely to be a limitation for this method. This may therefore restrict analysis of individuals to larger species. Even so, many of these species could not otherwise have reasonably been analysed using the existing multi‐collector ICP‐MS or TIMS methods with 58% of samples having >100 mg of Sr and only three samples in two of the larger species having >500 ng of Sr. At the very least, as most of the species show close to an order‐of‐magnitude variation in their Sr concentrations, it is advised that Sr concentration be measured prior to attempting isotope measurement. There are few reports of Sr measurements in insects, but one comparable study found similar Sr concentrations of *ca* 3–4 ppm for the larger grain borer, *Prostephanus truncatus* (3–4.5 mm long), although the samples were pooled insect specimens.^29^

Of the other factors, this study was not designed to test insect life style (habitat and diet) influences on Sr levels, but this and its potential influence on isotope ratios are now considered below as possible, with this method in mind for future research.

### 5.3 Ability to understand environmental and biological factors influencing variation in Sr isotopes

This study was not designed to assess the geographic differences of Sr isotopes, but a high degree of spatial variation was recorded in the individual insect specimens from metropolitan Brisbane (Figure 6). This could reflect the variation in the underlying geology given that the Sr isotopes analysed here involve two of the major rock types that occur in the metropolitan Brisbane region^30^: Tertiary volcanic rocks (^87^Sr/^86^Sr 0.7037 to 0.7107) and Carboniferous metasediments (^87^Sr/^86^Sr 0.7041 to 0.7187). Their Sr isotopes correspond to much of the range observed in the insects, but do not extend to the higher ^87^Sr/^86^Sr (>0.72) observed in some of the specimens. One explanation may be the paucity of Sr isotope measurements of geological samples from the region, although it is expected that rock types such as Carboniferous metasediments will have components with high ^87^Sr/^86^Sr of >0.72. Another explanation could be that the insect plant diet will be composed of Sr from a mix of sources in addition to the immediate geology, such as near‐time differences in ground and surface water. With generally very short life spans, except perhaps that of the brown cockchafer which lives for more than a year, short‐term events or seasonal perturbations may contribute to variation amongst individuals of a population. Similarly, the addition of horticultural products such as lime or fertiliser^31^ may explain the locations with variable ^87^Sr/^86^Sr such as the Brisbane Botanical Gardens.

The impact of various ecological and biological factors on intra‐population variation will now be more easily assessed without the need to pool specimens of many species. This will be useful to test if inter‐population variation for geographic places with different Sr isotope signatures is meaningful. For example, the different insect species in this study will have derived their composite Sr from across different geographic ranges. Some will have Sr isotopes from a very restricted areal extent, including the eucalyptus longhorn beetle larvae that live in just one tree, brown cockchafer larvae that do not move far in the soil, the common crow butterfly and sawfly ‘caterpillars’ that feed on one plant, and the gum tree and brown long‐headed shield bugs that are largely sedentary sap‐sucking insects. The blue banded and European honey bee during immature feeding life stages on the other hand are fed pollen from a variety of plants across a wider foraging area as is the predatory hornet on a diet of other insects from a potentially wide area. The Sr isotope signature of the latter class may be more complicated to interpret in terms of provenance if this involves plants on different geologies. However, this new method permitting use of single insects will enable factors such as these to be empirically tested where they could not have been before.

More fundamental aspects that may influence population variation will also be strengthened by the statistical analysis of Sr isotope data made possible by individual insect measurements. Examples are the influence of polyphagy, where the host plant species of the feeding immature life stage is unknown in the captured dispersing adult, and the effect of adult feeding on water or nectar as they disperse to places beyond their origin with potentially different geographic signatures from those of the immature life stages. The ability to use such low sample mass could also help to determine which adult tissues best retain the signature of the immature life stage through metamorphosis and therefore help to justify, or not, the preferential use of less metabolically active tissues such as exoskeleton structures. However, as the chemistry of Sr^+^ is similar to that of Ca^+^, such that Sr^+^ can metabolically substitute for Ca^+^ in tissues^2^, ^3^ and insects actively regulate their Ca^+^ to maintain homeostasis,^32^ the same mechanisms of sequestration and excretion probably also maintain a near homeostasis for Sr^+^. Any differences in how this is managed by different insect groups may have an influence on this.

### 5.4 Applications now possible with single‐insect Sr isotope measurements

The ability of this new ICP‐MS/MS method to measure Sr isotopes in single insects addresses the original driver for this research, to determine the origin of insects associated with a biosecurity incursion. In that case, there are typically few individuals of low mass, and operational speed and cost concerns. All these issues are favourably addressed by this method.

## 6 CONCLUSIONS

Strontium isotope analysis using ICP‐MS/MS with N_2_O as a reaction gas effectively converts Sr to SrO^+^ without appreciable Rb conversion to RbO^+^ during analysis. This allows for the ^87^Sr/^86^Sr to be determined from ^87^Sr^16^O/^86^Sr^16^O in Rb‐bearing samples without the need for prior chemical separation of Rb from Sr.

For the first time we show that, for Sr isotope analysis using ICP‐MS/MS, ^88^Sr^16^O/^86^Sr^16^O can be used to accurately correct for mass bias on ^87^Sr^16^O/^86^Sr^16^O. This gives an external reproducibility on mass bias‐corrected ^87^Sr/^86^Sr of 0.17% for the Sr carbonate NIST SRM 987 and 0.12% for NIST 1570a spinach leaves.

For viable Sr isotope analysis with a precision of >0.2%, only 2–4 ng of Sr is required for analysis using the ICP‐MS/MS method. For a conservative insect Sr concentration of 1 ppm this equates to 4 mg of insect tissue. This makes the techniques ideal for assessing the provenance of small quantities of biological material of biosecurity relevance.

For the insect species analysed here, individual insects show highly variable Sr concentration, but there is no significant variation in Sr concentration between different species. Insects that forage over a wide spatial area have much lower variation in ^87^Sr/^86^Sr than those that have a spatially restricted diet.

## Supporting information

Data S1 Supporting Information
